# Dietary Patterns of Adolescents from the Chilean Growth and Obesity Cohort Study Indicate Poor Dietary Quality

**DOI:** 10.3390/nu12072083

**Published:** 2020-07-14

**Authors:** Angela Martínez Arroyo, Camila Corvalán Aguilar, Ximena Palma Molina, Ximena Ceballos Sanchez, Regina Mara Fisberg

**Affiliations:** 1 School of Nutrition and Dietetics, Faculty of Pharmacy, University of Valparaíso, Valparaíso 2360102, Chile; angela.martinez@uv.cl (A.M.A.); ximena.palma@uv.cl (X.P.M.); ximena.ceballos@uv.cl (X.C.S.); 2 Department of Nutrition, School of Public Health, University of São Paulo, São Paulo 01246-904, Brazil; 3 Institute of Nutrition and Food Technology (INTA), University of Chile, Santiago 7830420, Chile; ccorval@gmail.com

**Keywords:** dietary pattern, adolescent diet, dietary quality, exploratory factor analysis

## Abstract

Diet during adolescence can have lasting effects on nutritional status, health, and development. We hypothesized that dietary patterns with low-quality nutrition are associated with overweightness. We collected data for 882 Chilean adolescents from the Growth and Obesity Cohort Study (mean age: 12 years). Dietary intake was assessed through 24-h recalls and weight status data were obtained during clinical visits. Dietary patterns were obtained through exploratory factor analysis. Multiple logistic regression models were used to examine cross-sectional associations between dietary patterns and overweight (BMI z-score ≥ 1 SD). Four dietary patterns were identified: “Breakfast/Light dinner”, “Natural foods”, “Western”, and “Snacking”. “Breakfast/Light dinner”, “Western”, and “Snacking” patterns provided higher energy and excess nutrients (sodium, saturated fat, and added sugar). Moreover, adolescents with higher adherence to “Western” or “ Snacking” patterns (third tertile) had higher odds of being classified as overweight (OR = 1.67; 95% CI: 1.103–2.522 and OR = 1.86; 95% CI: 1.235–2.792, respectively) than those with lower adherence (first tertile). “Natural foods” pattern was also associated with overweightness (OR = 1.83; 95% CI: 1.219–2.754). These dietary patterns were associated with overconsumption of nutrients of public health concern. Three of the four main dietary patterns were associated with overweightness. These results highlight the need of prioritizing adolescents on obesity prevention strategies.

## 1. Introduction

Adolescence is a critical stage in life, characterized by an accelerated growth rate and multiple physical and social changes that prepare a person for adulthood [1]. Diet quality during this period is important as it can affect adolescents’ nutritional status and can have long-lasting effects on their future feeding behavior, nutritional status, early life health, and the development of their offspring [2,3,4]. Evidence relating to diet during this period has been found in high-income countries (HIC) [1,2]. Nonetheless, in the regions of Latin America and the Caribbean (LAC), dietary aspects are not well studied. Thus, information on adolescents feeding habits are usually extrapolated from adult data findings, highlighting the need for further evidence [5].

The rising trend of overweightness and obesity in children and adolescents has plateaued in many HIC and industrialized low to middle-income countries (LMIC), albeit at high levels [6]. In the last few decades, in LAC, there has been a shift towards diets based mostly on foods with added sugar and salt, refined carbohydrates, grain-based desserts, and savory snacks [7]. In combination with a decline in physical activity, these diets have led to a rapid increase in childhood obesity in LAC countries [5,7,8].

Among LAC countries, Chile has one of the highest prevalence rates (up to 31%) of overweight and obesity in children and adolescents [5,8]. In addition, Chile has the highest measured per capita sales of sugar-sweetened beverages (SSBs), junk food, salted snacks, and foods that are high in added sugar, saturated fats, and sodium [7] among LAC countries.

There is now evidence that dietary patterns, rather than individual nutrients or foods, contribute to disease trajectories because empirical dietary patterns evaluate the food consumption under a multidimensional approach that takes into account the effects of the overall diet [9,10,11,12,13,14]. However, how the consumption of different foods is integrated within specific dietary patterns, particularly in LAC countries where there is great cultural and dietary diversity, is unclear [15]. Therefore, the objective of the current study is to characterize the dietary patterns of a cohort of low-middle income Chilean adolescents and to assess the associations of these diets with socio-demographic factors and excess body weight. Given that Chile is an LAC country with one of the highest obesity rates, we hypothesize that most common dietary patterns will be of low-quality nutrition (high in saturated fat, added sugars, and sodium) and will be associated with overweightness. Moreover, data were collected before the implementation of a set of regulatory actions to prevent obesity (warning labels, marketing, and control of food sold in schools) [16], and therefore, can serve as a baseline for assessing the effectiveness of the regulations among a critical age-group such as adolescents.

## 2. Materials and Methods

### 2.1. Study Population

Our study sample was obtained from adolescents participating in the Growth and Obesity Chilean Cohort Study (GOCS). Briefly, GOCS is a population-based ambispective cohort of 1195 children, born in 2002–2003, from six low-middle income counties from the southeast area of Santiago, Chile. Participants were children who were born at term (37–42 weeks), had a birth weight of between 2500 and 4500 g, and were free from conditions that could affect their growth, such as food allergies and genetic and metabolic diseases. Details of the design, objectives, and recruitment strategies of the GOCS have been described elsewhere [17,18]. GOCS contains data on anthropometric measurements and sexual maturation evaluations collected every six months since 2006 [19]. Dietary assessments were carried out in person during clinic visits in 2013 with the use of 24-h recalls (24HR).

**Figure 1:**
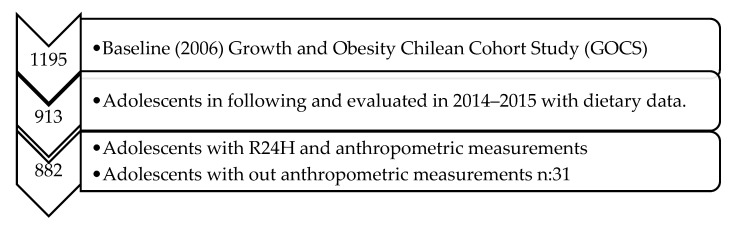
Flowchart for participant selection in the study.

The present study used data collected during the 8–9-year follow-up. In the present study (Figure 1), all adolescents were evaluated in 2014–2015, with at least one 24HR per participant (*n* = 913), and anthropometric measurements were obtained during the same clinic visit (*n* = 882). We excluded 31 adolescents (21 boys and 11 girls) because no anthropometric measurements were available. The final sample of this study included 882 adolescents. There were no differences in age and energy intakes between the total sample and the excluded participants (i.e., mean ages 12.07 vs. 12.02 years and energy intake 1828 vs. 1827 mean kilocalories/day, respectively).

The informed consent and the study protocol were approved by the Institutional Reviewer Board from the Institute of Nutrition and Food Technology, University of Chile. Before data collection, parents/caregivers provided written informed consent and adolescents provided written assent.

### 2.2. Dietary Data Collection

Dietary data were obtained through two non-consecutive 24HR interviews on different weekdays, weekends, and seasons, following the “Multiple Pass Method” [20]. About 60% of the second recalls were collected 6 months after the first recall. The interviews were conducted in person by trained dieticians in the presence of the person responsible for preparing and serving meals to the adolescents to avoid food misreporting. Dietary intake data included the time of the meal and between meals, names of dishes and cooking methods, and a list of food, beverages, and serving sizes (household measures), and was obtained with the aid of a photo atlas from the National Survey of Food Consumption of Chile [21]. Energy and nutrient intakes from 24HRs were calculated using the Nutrition Data System for Research (NDSR) program (version of 20014, NCC, University of Minnesota, Minneapolis, MN, USA), which uses the United States Department of Agriculture (USDA) database as the main food composition table. Therefore, all foods and beverages listed in the 24HR interviews were matched with those in the NDSR database through their general description (i.e., name, preparation, and cooking method). Energy and macronutrient values available in the Chilean Food Composition table [22], local food industry composition tables, and/or nutrition food labels were compared to values described by the software. A concordance rate between 80% and 120% for each parameter analyzed was required to accept food harmonization. Chilean preparations, such as charquicán, cazuela, and sopaipillas, were added as recipes to the NDSR because they are commonly consumed by Chilean adolescents and were not in the software database. We observed 24HRs with values over 3500 kcal and under 500 kcal [23], which were revised to rule out digitalization errors. We did not exclude any participant with these values.

In total, 1053 different foods were reported in two 24HRs, but 993 were consumed by at least 5% of the sample. These food items were collapsed into 29 food categories on the basis of their nutritional value, the coefficient correlation between groups, commonality values, food preparation methods, and Chilean dietary behaviors (Supplemental files Table S1).

### 2.3. Anthropometric Measurements

Anthropometric measurements were obtained using validated techniques, and they were duplicated by two trained dietitians using standard procedures (one for each sex, intra-, and inter-class correlation > 0.9) [19]. Bodyweight was measured with a standardized scale (TANITA BC-418) with 0.1 kg precision, and height was measured using a longitudinal stadiometer (SECA 222) ranging from 6 to 230 cm in capacity and a precision of 0.1 cm. Body mass index (BMI) was defined using the standard formula: weight (kg) divided by height squared (m^2^). The BMI z-score was calculated according to the World Health Organization (WHO) definition for age and sex, and it was used to classify the adolescents’ body weight status as underweight (≤−1 SD), normal weight (<−1 SD and <+1 SD), overweight (≥+1 SD and <+2 SD), and obese (≥+2 SD) [24]. We used the term overweight (outcome variable) when referring to overweight and obesity nutritional status combined.

### 2.4. Covariates

#### 2.4.1. Sexual Maturation

A pediatric endocrinologist evaluated the adolescents’ breast and genital development and classified them according to the Tanner stages. Girls were evaluated according to their breast development through inspection and palpation using the Tanner scale. Boys were evaluated according to their genitalia development through palpation using the Prader orchidometer [25].

#### 2.4.2. Maternal Self-Reported Information

The following information was obtained: (i) maternal highest education level (≥12 y or <12 y), (ii) children’s school administrative status (private or public), (iii) hours of sleep (meeting or not meeting the recommendations ≥ 9 h/day or < 9 h/day), and (iv) participation in the school feeding program (yes or no).

The school feeding program aims to provide daily food services (breakfast, lunch, light dinner (a typical Chilean meal served sometime between 5:00 and 8:00 pm, comparable to teatime), snacks, and dinner, as appropriate) to all students from vulnerable sections of society in Chile’s public schools. In the present study, 30% of the adolescents were reported to be beneficiaries of the school feeding program.

#### 2.4.3. Maternal Weight Status

A trained dietitian measured the participants’ mothers’ weights and heights to calculate their BMI and classify their weight status according to the WHO guidelines [26].

### 2.5. Statistical Analysis

Firstly, we used statistical modeling techniques incorporated into the Multiple Source Method online platform, which were used to estimate the usual intake of each food group in grams [27]. Exploratory factor analysis (EFA) was used to identify the dietary patterns from 29 food groups. Initially, factors with eigenvalues greater than or equal to 1.5 were retained. In the second stage, the scree plot was visually inspected, and it suggested the retention of four factors. The Varimax orthogonal rotation was applied for better interpretation of the factor loading matrix and to ensure the independence of the factors derived. Factor loadings greater than or equal to |0.25| were considered to contribute to the pattern [28]. The retained patterns were named according to their interpretability, the characteristics of the food in each dietary pattern, and the commonalities observed, which reflect the level of linkage between the variable (food group) and the extracted factor. Other food grouping systems were tried but they did not allow us to create dietary patterns because of the low factor loading and their interpretability. For each participant, the factor score of each dietary pattern was calculated using the regression scoring method [29]. The dietary pattern scores were then stratified into tertiles and used as independent variables. The χ^2^ Pearson’s test was used to investigate the associations between the tertile adherence to dietary patterns and weight status, Tanner stages, and socio-demographic and maternal variables (bivariate analyses).

We also used the Multiple Source Method to estimate the usual intake of energy and critical nutrients of public health concern (i.e., sodium, added sugars, and saturated fats) [30]. Individual usual nutrient intakes were expressed in grams or micrograms per 1000 kcal of energy, or as total percentage of energy intake.

Medians and interquartile ranges were provided according to factor score tertiles. The associations between usual nutrient intakes and adherence to dietary patterns were calculated by applying the Kruskal–Wallis and Dunn tests.

Misreporting of energy intake was estimated with the following equation: EI (energy intake) − EER (estimated energy requirements)/EER × 100 [31]. Misreporting was differentially associated to the dietary patterns (i.e., breakfast/light dinner *r* = 0.02, natural foods *r* = 0.02, Western *r* = 0.14, snacking *r* = 0.15); therefore, this variable was adjusted for in all regression models. Logistic regression models were used to investigate the association between adherence (tertiles) to each dietary pattern and overweight (dummy variable defined herein as overweight [yes or no]) with covariates adjusted. All analyses were performed using STATA software version 15 (College Station, TX 77845) with significance values established for all tests at *p* < 0.05 (5%).

## 3. Results

### 3.1. Demographics and Lifestyle Characteristics

The sample from GOCS included 882 respondents (mean age 12 years, SD = ±0.7), of which 50.8% were female. The majority of the adolescents were classified as overweight or obese (51.6%). Almost half of the participants (49.7%) were classified into the fourth and fifth Tanner stages of sexual maturation. More than 60% of the participants slept for more than 9 h/day and studied at private schools and one third were beneficiaries of the school feeding program. In relation to the maternal socio-demographic and weight status, over half (65.5%) of the participant mothers did not have more than 12 years of study, and 38.3% were classified as obese (Table 1).

### 3.2. Identification of Dietary Patterns

Exploratory factor analysis allowed the identification of four dietary patterns: (i) “Breakfast/Light Dinner”, consisting of tea, sugar, bread, margarine/butter, and cold cuts (positive loadings); (ii) “Natural foods”, consisting of meats, vegetables, and salad dressing (positive loadings); (iii) “Western”, consisting of processed meats, soft drinks, rice, pastas, potatoes, mayonnaise, ketchup (positive loadings) and milk, homemade dishes (typical Chilean recipes, such as charquicán, carbonada, pastel de choclo, cazuela), and chocolate powder (negative loadings); and (iv) “Snacking”, consisting of flavored milk, cookies, and cakes (positive loadings), and yoghurts and ready-to-eat cereals (negative loadings). These patterns resulted in a total intake variance of 7.4%, 6.4%, 5.6%, and 5.4%, respectively (Table 2).

### 3.3. Adherence to Dietary Patterns, Socio-Demographics, Weight Status, and Maternal Variables

The frequency of adherence (%) to dietary patterns and the bivariate relationships with socio-demographics and weight status, i.e., pubertal and maternal weight statuses, are shown in Table 3. Adherence to the “Breakfast/Light dinner” pattern was higher among underweight and obese adolescents (*p* = 0.01). Adherence to the “Natural foods” pattern was higher among adolescents who were not recipients of the school feeding program (*p* = 0.04). The adherence to the “Western” pattern was higher among male adolescents (*p* = 0.04) and those who did not participate in the school feeding program (*p* = 0.04). Adherence to the “Snacking” pattern was higher in adolescents that partook in the school feeding program (*p* = 0.01).

### 3.4. Nutrient Intake and Adherence to Dietary Patterns

Associations between nutrient intake and adherence to dietary patterns are shown in Table 4. Adolescents with higher adherence to the “Breakfast/Light dinner”, “Western”, and “Snacking” patterns (third tertile) had a higher energy intake than those who showed lower adherence (first tertile). The contribution of saturated fats was significantly higher in the “Western” pattern, whereas added sugars accounted for a significantly higher percentage of energy contribution for adolescent “Snacking” consumers. The “Natural foods” and “Breakfast/Light dinner” patterns were associated with lower intakes of saturated fats but a higher intake of sodium.

### 3.5. Adherence to Dietary Patterns and Overweight

When adjusted for covariates (i.e., sex, age, Tanner status, misreporting, maternal obesity, and education level), adolescents with higher adherence to the “Natural foods”, “Western”, and “Snacking” patterns had higher odds of being classified as overweight than those with lower adherence (OR = 1.83, 95% CI: 1.21–2.75; OR = 1.67, 95% CI: 1.10–2.52 and OR = 1.86, 95% CI: 1.23–2.79, respectively). The “Breakfast/Light dinner” pattern was not significantly associated with overweightness (Table 5).

### 3.6. Sensitivity Analyses

We tested other combinations of food groups that had low factor loadings (e.g., we combined the eggs and cheeses groups, and confectionary, desserts, and ice cream groups, for their commonalities) but their communalities and factor loadings did not improve; indeed, the consistency of the findings remained the same (data not shown).

## 4. Discussion

The present study identified four major dietary patterns among Chilean adolescents: “Breakfast/Light dinner”, “Natural foods”, “Western”, and “Snacking” patterns. All dietary patterns identified in this sample were associated with either a high caloric intake or overconsumption of a nutrient of public health concern, such as saturated fats and added sugars or sodium. Moreover, the “Western” and “Snacking” patterns showed a positive association with overweightness; however, the “Natural foods” pattern was also associated with overweightness; these adolescents may have been consuming these foods in an attempt to combat their health problems.

In this population, the “Breakfast/Light dinner” pattern showed the highest percentage of dietary variance. This pattern describes a very typical diet of the Chilean population [21]; it accounts for two of the four main meals in Chile, i.e., breakfast, lunch, “Light dinner ” (similar to breakfast in terms of food consumed, but it is taken at 5:00–8:00 pm) and dinner. In Chile, it has been recently observed that dinner is being increasingly replaced by “Light dinner” and, currently, only one in four Chileans over 18 years of age eat dinner [21]. This may have a potential effect on the shortfall of quality nutrients, as dinner in some cultures often includes a variety of healthy meats, vegetables, integral cereals, and fruits [32]. In this study, adolescents with a higher adherence to the “Breakfast/Light dinner” pattern had higher energy and sodium intakes, and obese adolescents showed a higher adherence to this pattern. Bread, which is one of the main components of this pattern and a major source of energy and sodium intake, is the most consumed food among Chileans (i.e., 86.5 kg/person/year), particularly among low-income individuals [21,33,34]. The Latin American Study of Nutrition and Health also observed that refined-grain products, such as bread, are a major source energy in Latin American countries; however, Chile had a higher percentage (25.13%) of energy contribution through refined-grains than Colombia (11.71%) [35], which has a lower obesity prevalence in adolescents [8].

Studies have shown that dietary patterns with high factor loadings, e.g., the bread group, have higher energy densities and positive associations with overweightness [36,37,38]. We found that foods groups of this pattern (white bread, sugar, margarine, cold cut, and tea) provide low quality nutrition, similar to “traditional breakfast” patterns found by Oliveira et al. in Brazilian adults [39]; however, we did not find this food group to be associated with overweightness. This might require further study, as our sample was homogenous in terms of socioeconomic status (SES), and this pattern is a dietary behavior typical of the Chilean population [21]. However, our findings that this pattern explained the higher variability of diet is worrying, because adolescence is a stage critical for rapid growth, and to maintain this behavior could lead to negative health consequences in adult life [4].

The “Natural foods” pattern was the second most relevant in this population. This pattern could be considered healthier because it has a higher factor loading of natural foods, such as vegetables and meats. Furthermore, this pattern was more frequent among those not participating in the school feeding program (based on SES) and whose mothers had a higher education level. The latter is in agreement with previous reports that high SES individuals have healthier dietary behaviors than their counterparts with lower SES [21]. In addition, in line with the literature, we found an inverse association between adherence to the “Natural foods” dietary pattern and energy intake [12,37,40]. In contrast with this finding and that of other studies that show a likely protective role against obesity [15,41], we found that the “Natural foods” pattern was associated with a higher odds of being classified as overweight. Cross-sectional studies in Mexican adolescents [42] and Norwegian children also have found a positive association between higher adherence to a healthy dietary pattern and overweightness [43,44]. We believe this is explained by reverse causality [45], whereby overweight adolescents have either already changed their diet to a more healthy one before the assessment or they under-report unhealthy food and over-report healthy food [46].

The “Natural foods” pattern was mainly composed of natural foods, and we did not expect it to be associated with high sodium and low saturated fat intakes. Negative factor loadings of processed meats, cold cut, and junk food may explain the low intake of saturated fat, and the addition of salt (higher factor loadings of oil, lemon, salt, and vinegar foods) to salads, which is in line with recent analyses of the National Survey of Food Consumption [21], could also explain the high sodium intake. These results are different from those found for American children and adolescents, where the main sources sodium were pizza, Mexican dishes, sandwiches, breads, cold cuts, soups, savory snacks, etc. [47]. Assessing sodium intake using 24HR is a challenge for epidemiological studies, because intake is likely to be incorrectly estimated or not accurately recalled. We used household measures together with a photo atlas [21] and up-to-date labelling of food to aid in the accurate estimation of sodium intake. These results highlight the need for consumer awareness and educational activities, in conjunction with other salt-reduction strategies, to address this public health problem [48].

“Western” and “Snacking” patterns were characterized by unhealthy and processed foods of low nutritional quality, with the exception of the meats group and the rice, pastas, and potatoes group, which are good sources of protein and carbohydrates and are staple Chilean foods [21]. It is important to note that the “Western” pattern was characterized by a high negative loading of the homemade dishes group (homemade dishes or healthy meals consisting of potatoes, carrots, corn, pumpkin, and red meat or poultry stews), which could indicate that adolescents who have high scores for this pattern consume more ready-to-eat or ready-to-heat foods than vegetable soups and legume stews with proteins (animal or vegetable). Such dishes require a longer cooking time but are nutritionally balanced and culturally appropriate for adolescents, according to Chilean dietary guidelines [32,49]. These results are consistent with the evidence for the increase in expenditure on ready-to-eat meals, SSBs, and away-from-home foods in Chile in recent years [50,51]. There is evidence that consumption of SSBs promotes weight gain in children and adults; therefore, discouraging the consumption of SSBs is an important way to help children achieve and maintain a healthy body weight [52]. As expected, adolescents with higher adherence to the “Western” and “Snacking” patterns had higher energy intakes, although the nutrient sources differed between these patterns, i.e., saturated fat for the “Western” (from processed meat and cold-cuts) and added sugars (from flavored milk, cookies, and cakes) for the “Snacking” patterns. These findings are in line with dietary patterns high saturated fat and added sugar found for Australian adolescents [37]. According to a systematic review on empirical dietary patterns [41] and other studies in Brazil [36], Mexico [42], and Colombia [53], dietary patterns named “Snack” and “Western” (characterized by processed foods of low nutritional quality) were also associated with an increased risk of overweight in children and adolescents; these results are in line with our findings. This is important because the objective of front labeling and the control of food marketing aimed at children in Chile is to regulate the consumption of unhealthy food groups (including processed meat, soft drinks, cookies, and cakes) and to inform consumers, through a warning symbol on packaged products, that they are high in energy, saturated fats, total sugars, or sodium [16].

Dietary patterns were, in general, homogeneously distributed among the study sample. We observed that boys had higher adherence scores for “Western” patterns than girls, probably because girls under-report these types of foods, and because girls are more vulnerable to social aesthetic standards [54]. We also observed that participation in the school feeding program protected adolescents from the “Western” dietary pattern, probably because this program provides beneficiaries with a free breakfast and lunch that includes fresh fruits and vegetables, as well as fulfilling nutrient requirements based on dietary guidelines [49,55]. However, participation in the school feeding program did not protect individuals from the “Snacking” pattern, which is probably because snacks are also available inside schools, in addition to main meals [56].

The limitations of the study should be considered when interpreting its results. First, its cross-sectional design limits us from establishing causality. Besides, the present study used EFA, a method that involves decision-making by researchers at various stages of the modeling process, such as decisions on grouping and the number of factors to be selected. For this reason, widely-used nutritional epidemiology procedures were applied and validated to counterbalance these weaknesses [57] and several sensitivity analyses confirmed the validity of our findings. Empirical dietary patterns usually differ across studies. For that reason, we tried to label the found dietary patterns with the same nomenclature than other studies [14]. However, we also considered the way the Chilean population eats the foods included in the patterns to reflect their habitual meals. Although we used 24HRs, which might be subject to recall bias and do not estimate the usual dietary intake, 61% of the sample underwent a second measurement within 6 months. This allowed us to estimate the usual intake, adjusting for intra-individual variance according to the Multiple Source Method, and to increase the reliability of the results [27]. The interviews were reported by parents or caregivers who had access to the school feeding program menus, which complemented the dietary data and followed the “Multiple-Pass Method.” The study population was somewhat homogeneous in terms of SES, and the inclusion of more variety may have had an effect on the differences between groups [58]. Analyses of the adherence to dietary patterns and overweight status were not adjusted for physical activity due to a lack of information, but it was possible to adjust the final models for the Tanner pubertal stage (i.e., a relevant confounding factor among adolescents) [1]. Finally, this study considered adjusting the regression models by misreporting to account for possible measurement errors associated with self-reported dietary data (R24H), which could impact the obtained dietary patterns, and the association with overweight [46].

## 5. Conclusions

In a sample of low-middle income adolescents from Chile, a country with one the highest prevalence of obesity worldwide, we found that common dietary patterns are related to high consumption of calories and nutrients of public health concern were associated with overweightness, confirming the hypothesis of this study. We did not identify dietary patterns that could be considered healthy, indicating that intake of healthy foods among this age group is random and does not conform to an organized pattern. Our results are of concern and demonstrate poor dietary quality during a period that can have long-lasting implications for both the individual and their potential offspring. It is important to evaluate whether the ongoing obesity prevention policies will be able to modify these unhealthy behaviors among a traditionally resistant age-group.

## Figures and Tables

**Table 1:** Anthropometric, sociodemographic, and maternal characteristics of 882 Chilean adolescents, GOCS Study 2014–2015.

Characteristics	*n*	%
Sex		
Male	434	49.2
Female	448	50.8
Tanner Stage ^¥^		
1	54	6.1
2	222	25.2
3	144	16.3
4	257	29.1
5	182	20.6
Missing	23	2.6
Weight status (BMI-for-age *z* score) *		
Underweight	43	4.9
Normal Weight	383	43.5
Overweight	275	31.2
Obesity	180	20.4
Sleep (hours)		
≥9 h	236	26.8
<9 h	532	60.3
Missing	114	12.9
School administrative status		
Private	543	61.6
Public	330	37.4
Missing	9	1
School feeding program		
No	603	68.4
Yes	279	31.6
Maternal Obesity ^†^		
No	520	59.0
Yes	338	38.3
Missing	24	2.7
Maternal education (years)		
<12 years	578	65.5
≥12 years	280	31.8
Missing	24	2.7

* BMI-for-age *z* score: underweight ≤ −1 SD, normal < −1 SD and < +1 SD, overweight ≥ +1 SD and < +2 SD, obesity ≥ +2 SD [24]; † Maternal obesity = BMI ≥ 30 kg/m^2^. ¥ Tanner status: girls were evaluated according to their breast development and boys were evaluated according their genitalia development [25].

**Table 2:** Dietary Patterns practiced by GOCS adolescents (*n*:882), Chile 2014–2015.

Food and Beverage Groups	Breakfast/Light Dinner	Natural Foods	Western	Snacking
Milk	−0.22	0.00	−0.32	−0.12
Flavored milk	−0.24	−0.20	0.06	0.28 *
Yogurt	−0.10	0.03	0.01	−0.73 *
Cheeses	0.06	−0.06	0.16	0.11
Meats	−0.16	0.50 *	0.29 *	0.07
Cold cuts	0.27 *	−0.15	0.27 *	0.00
Processed meats	0.01	−0.20	0.41 *	−0.07
Junk Food	−0.18	−0.23	0.11	0.09
Flavored juices	−0.22	0.16	−0.07	0.24
Soft drink	−0.07	0.03	0.41 *	0.14
Tea and coffee	0.73 *	0.04	−0.11	0.08
Bread	0.64 *	−0.04	0.18	0.11
Ready to eat cereal	−0.09	0.03	−0.02	−0.77 *
Rice, pastas, potatoes	0.02	0.16	0.47 *	−0.05
Vegetables	0.09	0.81 *	−0.01	0.00
Fresh fruit	0.08	0.16	−0.23	0.05
Eggs	0.02	0.04	0.00	−0.07
Homemade dishes	0.14	−0.11	−0.62 *	0.01
Soups	−0.10	0.18	−0.19	0.13
Cracker and salt snack	−0.14	−0.11	0.16	0.03
Confectionery and chocolate	−0.07	0.02	−0.03	0.17
Cookies	−0.23	−0.07	−0.11	0.29 *
Cakes	−0.09	0.03	−0.19	0.25 *
Desserts and ice cream	0.05	0.08	0.01	−0.03
Sugar	0.70 *	0.10	−0.12	0.08
Chocolate powder	−0.19	−0.03	−0.27 *	0.01
Margarine and butter	0.49 *	−0.08	0.05	−0.09
Oil, lemon, salt, vinegar (for salad)	0.00	0.79 *	0.03	−0.07
Mayonnaise and ketchup	−0.01	0.04	0.31 *	0.06
% of explained variance	7.42	6.42	5.64	5.43
% of accumulated explained variance	7.42	13.84	19.48	24.90
Eigenvalues	2.16	1.88	1.63	1.55

* All Factor loadings |0.25| are shown in bold for readability purposes. Bartlet sphericity test = *p* < 0.0001, KMO: 0.52.

**Table 3:** Adherence (%) according to sociodemographic, anthropometric, and maternal nutrition status of 882 Chilean adolescents, GOCS Study 2014–2015 Chile.

**Characteristics**		**Breakfast/Light Dinner**							**Natural Foods**						
		**T1**		**T2**		**T3**		***p***	**T1**		**T2**		**T3**		***p***
		**%**	***n***	**%**	***n***	**%**	***n***		**%**	***n***	**%**	***n***	**%**	***n***	
Sex	Male	30.2	131	34.1	148	35.7	155	0.126	35.5	154	33.4	145	31.1	135	0.292
	Female	36.4	163	32.6	146	31.0	139		31.3	140	33.3	149	35.5	159	
Tanner stage ^¥^	1–3	31.4	132	33.6	141	35.0	147	0.617	36.0	151	32.9	138	31.2	131	0.243
	4–5	34.4	151	33.0	145	32.6	143		30.8	135	34.2	150	35.1	154	
Weight status ^‡^	Underweight	30.2	13	23.3	10	46.5	20	0.013 *	32.6	14	32.6	14	34.9	15	0.167
	Normal Weight	36.2	139	30.0	115	33.9	130		37.0	142	34.6	133	28.4	109	
	Overweight	33.1	91	40.0	110	26.9	74		31.3	86	33.1	91	35.6	98	
	Obesity	28.3	51	32.8	59	38.9	70		28.9	52	31.1	56	40.0	72	
Sleep Hours	≥9 h	33.5	79	34.8	82	31.8	75	0.727	32.2	76	37.3	88	30.5	72	0.324
	<9 h	35.7	190	32.0	170	32.3	172		34.6	184	31.8	169	33.7	179	
School administrative status	Public	30.6	101	32.4	107	37.0	122	0.186	34.9	115	32.7	108	32.4	107	0.728
	Private	34.8	189	34.1	185	31.1	169		32.2	175	34.1	185	33.7	183	
School feeding program ^£^	Yes	32.6	91	33.3	93	34.1	95	0.939	37.3	104	35.1	98	27.6	77	0.042 *
	No	33.7	203	33.3	201	33.0	199		31.5	190	32.5	196	36.0	217	
Maternal Obesity	Yes	36.5	100	32.7	113	30.8	125	0.069	33.3	113	33.1	114	33.7	111	0.966
	No	29.6	190	33.4	170	37.0	160		33.4	173	33.7	172	32.8	175	
Maternal education years	≥12 years	35.3	204	32.7	189	32.0	185	0.304	31.8	184	32.9	190	35.3	204	0.076
	<12 years	30.0	84	35.7	100	34.3	96		38.2	107	33.6	94	28.2	79	
**Characteristics**		**Western**							**Snacking**						
		**T1**		**T2**		**T3**		***p***	**T1**		**T2**		**T3**		***p***
		**%**	***n***	**%**	***n***	**%**	***n***		**%**	***n***	**%**	***n***	**%**	***n***	
Sex	Male	29.3	127	35.7	155	35.0	152	0.040 *	33.9	147	31.8	138	34.3	149	0.627
	Female	37.3	167	31.0	139	31.7	142		32.8	147	34.8	156	32.4	145	
Tanner status ^¥^	1–3	32.4	136	36.0	151	31.7	133	0.324	33.3	140	32.9	138	33.8	142	0.900
	4–5	33.9	149	31.2	137	34.9	153		33.9	149	33.7	148	32.4	142	
Weight status ^‡^	Underweight	27.9	12	41.9	18	30.2	13	0.675	34.9	15	27.9	12	37.2	16	0.408
	Normal Weight	35.4	136	30.5	117	34.1	131		35.9	138	30.5	117	33.6	129	
	Overweight	31.6	87	34.2	94	34.2	94		33.1	91	34.2	94	32.7	90	
	Obesity	32.8	59	36.1	65	31.1	56		27.8	50	39.4	71	32.8	59	
Sleep Hours	≥9 h	33.9	80	32.2	76	33.9	80	0.663	33.5	79	31.4	74	35.2	83	0.611
	<9 h	32.7	174	35.5	189	31.8	169		34.8	185	33.7	179	31.6	168	
School administrative status	Public	34.6	114	31.5	104	33.9	112	0.670	31.5	104	37.9	125	30.6	101	0.082
	Private	32.8	178	34.4	187	32.8	178		34.4	187	30.6	166	35.0	190	
School feeding program ^£^	Yes	36.9	103	35.5	99	27.6	77	0.046 *	27.6	77	32.3	90	40.1	112	0.007 *
	No	31.7	191	32.3	195	36.0	217		36.0	217	33.8	204	30.2	182	
Maternal obesity	No	35.2	183	33.7	175	31.2	162	0.197	33.1	172	34.2	178	32.7	170	0.719
	Yes	30.2	102	33.4	113	36.4	123		33.7	114	31.7	107	34.6	117	
Maternal education years	≥12 years	33.0	191	35.3	204	31.7	183	0.265	35.1	203	32.5	188	32.4	187	0.561
	<12 years	33.2	93	30.4	85	36.4	102		31.4	88	34.6	97	33.9	95	

T: Tertile adherence. All values are shown as percentages (for categorical). ¥ Tanner status: girls were evaluated according to their breast development and boys were evaluated according their genitalia development [25]. ‡ Nutritional status, BMI-for-age *z* score: underweight ≤ −1 SD, normal < −1 SD and < +1 SD, overweight ≥ +1 SD and < +2 SD, obesity ≥ +2 SD [24]. £ School feeding program: adolescents receive some food services of feeding program (yes or no). * X^2^ Pearson tests, *p* value < 0.05.

**Table 4:** Medians nutrient intake according to tertile ^†^ of adherence to dietary patterns in GOCS adolescents (*n*:882).

Dietary Patterns		Saturated Fat (%) IE ^§^			Added Sugars (%) IE ^§^			Sodium mg/1000 kcal			Energy/day (kcal)		
		Medians	IQR ^†^	*p* ^‡^	Medians	IQR ^†^	*p* ^‡^	Medians	IQR ^†^	*p* ^‡^	Medians	IQR ^†^	*p* ^‡^
Breakfast/Light dinner	tertile 1	9.8	1.8	0.001 ^abc^	18.3	6.6	0.285	1468	286	0.001 ^ab^	1754	504	0.017 ^c^
	tertile 2	9.6	2.1		17.4	6.8		1535	261		1758	464	
	tertile 3	9.0	2.4		17.3	6.4		1515	254		1819	427	
Natural foods	tertile 1	9.7	2.2	0.001 ^bc^	17.5	6.5	0.949	1478	257	0.017 ^ab^	1809	478	0.194 ^c^
	tertile 2	9.6	2.2		17.9	6.3		1521	286		1763	442	
	tertile 3	9.1	2.1		17.4	7.3		1530	277		1781	464	
Western	tertile 1	9.5	2.0	0.077 ^bc^	17.5	6.8	0.404	1488	304	0.424 ^b^	1738	441	0.001 ^bc^
	tertile 2	9.4	2.2		18.2	7.3		1514	272		1733	429	
	tertile 3	9.7	2.5		17.2	6.2		1526	247		1913	417	
Snacking	tertile 1	9.6	2.1	0.215 ^a^	16.5	6.5	0.001 ^bc^	1528	263	0.001 ^bc^	1704	470	0.001 ^bc^
	tertile 2	9.5	2.3		16.8	6.5		1553	288		1887	446	
	tertile 3	9.5	2.6		19.3	6.2		1460	258		1754	504	

Tertile 1 lower adherence, tertile 3 higher adherence. § IE, energy intake day. † Interquartile range (IQR). ^a^ Significant difference between tertile 1 and 2 of the factor scores. ^b^ Significant difference between tertile 1 and 3 of the factor scores. ^c^ Significant difference between tertile 2 and 3 of the factor scores. ‡ Kruskal–Wallis test and Dunn’s test post hoc, *p* value < 0.05.

**Table 5:** Crude odds ratio and adjusted odds ratio of overweight, according to tertile of dietary pattern practiced by GOCS adolescents. Chile, 2014–2015.

Dietary Patterns	Crude Model			Adjusted Model 1 ^§^			Adjusted Model 2 ^|^		
	ORs ^†^	95% CI *		ORs ^†^	95% CI *		ORs ^†^	95% CI *	
**Breakfast/Light dinner**									
tertile 1 ^¶^	Ref ^‡^			Ref			Ref		
tertile 2	1.45	1.045	2.003 *	1.28	0.903	1.815	1.25	0.839	1.875
tertile 3	1.03	0.744	1.420	0.90	0.634	1.274	1.03	0.689	1.529
**Natural foods**									
tertile 1	Ref			Ref			Ref		
tertile 2	1.13	0.818	1.562	1.15	0.810	1.620	1.07	0.717	1.583
tertile 3	1.55	1.119	2.146 *	1.68	1.180	2.390 *	1.83	1.219	2.754 *
**Western**									
tertile 1	Ref			Ref			Ref		
tertile 2	1.19	0.864	1.651	1.14	0.802	1.608	1.07	0.720	1.603
tertile 3	1.06	0.764	1.459	1.00	0.701	1.418	1.67	1.103	2.522 *
**Snacking**									
tertile 1	Ref			Ref			Ref		
tertile 2	1.39	1.003	1.920 *	1.53	1.077	2.160 *	1.51	1.013	2.235 *
tertile 3	1.12	0.807	1.541	1.19	0.839	1.680	1.86	1.235	2.792 *

* 95% Confidence Intervals, value *p* < 0.05. † ORs: odds ratio, CI: confidence interval. ‡ Ref: reference. § Logistic model 1: adjusted for sex, age, tanner, maternal obesity, maternal education (*n*:816). | Logistic model 2: adjusted for sex, age, tanner, maternal obesity, maternal education and misreporting (*n*:816). ^¶^ Tertile 1 lower adherence, tertile 3 higher adherence.

## Supplementary Materials

The following are available online at https://www.mdpi.com/2072-6643/12/7/2083/s1, Table S1: Description of the foods that composed each of the 29 food groups included in the factor analysis. GOCS Study, Chile 2014–2015.
