# Supplementary material for: Dietary Patterns of Adolescents from the Chilean Growth and Obesity Cohort Study Indicate Poor Dietary Quality
Source: Nutrients. 2020 Jul 14;12(7):2083. doi: 10.3390/nu12072083 (PMC7400834; doi:10.3390/nu12072083)
Supplement: Supplementary file 1 [file nutrients-12-02083-s001.pdf]

Supplemental Files

Table S1. Description of the foods that composed each of the 29 food groups included in the factor analysis. GOCS Study, Chile 2014–2015.

**Food Groups**	**Food Composition**
Milk	Fluid whole milk (3% fat), whole milk powder, reduced fat milk (2% fat), skim milk, skim milk powder
Flavored milk	Mixtures and milk drinks, strawberry, chocolate and other flavors, purchased ready-to-drink
Yogurts	Yogurt, fermented milk
Cheeses	Gouda cheese, ricotta cheese, cream cheese, cheddar cheese,
Meat	Steak beef, ground beef, beef ribs, pork chop, pork ribs, pork loin, poultry, chicken, turkey, fish, viscera (all cooking methods)
Cold cuts	Ham, bologna, turkey breast, chicken breast, salami
Processed meats	Sausage, sausages, frankfurters, meatballs ready for consumption, nuggets, hamburger ready for consumption (only meat).
Junk food	Pizzas, sandwiches of meat or hamburger ready to eat, french fries, wonton, egg roll, chilean hot dog, *sopaipillas, tacos, empanadas*. Mainly, street food or fast food
Sweetened beverages	Juice or flavored drink, purchased ready-to-drink, juice or flavored drink, dry mix - unprepared
Soft drinks	Soda pop or soft drink regular or diet
Coffee and tea	Coffee, instant coffee, herbal tea, tea bag
Bread	French bread, bun bread, white bread with salt
Ready to eat cereal	Breakfast cereals
Rice, potato and pasta	Rice, cooked potatoes, mashed potatoes, pasta and dishes pasta.
Vegetables	Lettuce, cabbage, raw salad, others load vegetables. Pumpkin, carrot, cucumber, tomato, among others.
Fruits	Pineapple, banana, orange, apple, pear, papaya, mango, watermelon, tangerine, grape, blueberry, strawberry, blackberry, fruit salad, fruit juices
Eggs	Fried eggs, scrambled eggs, omelet, boiled eggs, egg white, egg yolk
Homemade dishes	Typical chilean food or meals prepared at home, restaurants or schools; that require a longer preparation time and made with natural foods. For instance: Beans, lentils, chickpeas, white beans, legumes-based preparations, vegetables stews with or without meat, *cazuela, charquican, chapsui, humitas, pastel de choclo* among others.
Soup	Dry soup, bouillon, consommé.
Cracker and salt snack	Crackers, saltine or soda, salty chips - snack type, cheese balls, puffs or twists, potato chips
Chocolates and confectionary	Chocolate candy, chocolate candy bar, sweets based on milk, lollipop, candy, caramel, jams, *dulce de leche*

Cookies	Cookies and bars, granola bars, sweet biscuit and cookie stuffed, cookie sandwich, cookies commercial packaged
Cake	Cakes, cheesecake, cake sponge, doughnut, muffins, pies fruit, cupcake, cake purchased ready-to-eat
Desserts and ice cream	Pudding, flan, mousse, gelatin dessert, chilean desserts, fruit canned with syrup, ice cream and frozen desserts, Popsicle
Sugar	White sugar
Chocolate powder	Cocoa powder, chocolate powder
Butter and margarine	Salted butter, unsalted butter, salted margarine, unsalted margarine, light margarine
Oil, lemon, salt, vinegar (for salad)	Soybean oil, sunflower oil, vegetal oil, olive oil, salt, vinegar, lemon juice to salad
Mayonnaise, ketchup	Mayonnaise or mayo type dressing, ketchup, mustard, soy sauce
